# Explaining culturally and linguistically diverse (CALD) parents’ access of healthcare services for developmental surveillance and anticipatory guidance: qualitative findings from the ‘Watch Me Grow’ study

**DOI:** 10.1186/s12913-017-2143-1

**Published:** 2017-03-22

**Authors:** Pankaj Garg, My Trinh Ha, John Eastwood, Susan Harvey, Sue Woolfenden, Elisabeth Murphy, Cheryl Dissanayake, Bin Jalaludin, Katrina Williams, Anne McKenzie, Stewart Einfeld, Natalie Silove, Kate Short, Valsamma Eapen

**Affiliations:** 1 Department of Community Paediatrics, Liverpool Hospital, Liverpool, Australia; 2 School of Women’s and Children’s Health, UNSW, Liverpool, Australia; 3 School of Medicine, University of Sydney, Westmead, Australia; 4 Ingham Institute of Applied Medicine, Liverpool, Australia; 5 University of Western Sydney, Penrith, NSW Australia; 6 School of Women’s and Children’s Health, University of New South Wales, Sydney, NSW Australia; 7 School of Public Health, University of Sydney, Sydney, NSW Australia; 8 Ingham Institute of Applied Medicine, Liverpool, Australia; 9 School of Medicine, Griffith University, Gold Coast, QLD Australia; 10 Syney Local Health District, Croydon, NSW Australia; 11 School of Nursing and Midwifery, Griffiths University, Queensland, Australia; 12 Sydney Children’s Hospital Network (Randwick), UNSW, Sydney, Australia; 13 NSW Ministry of Health, Sydney, Australia; 14 Olga Tennison Autism Research Centre, La Trobe University, Bundoora, Victoria Australia; 15 South Western Sydney Local Health District and UNSW, Sydney, Australia; 16 Royal Children’s Hospital, Melbourne and University of Melbourne, Murdoch Childrens Research Institute, Melbourne, Australia; 17 Primary and Community Health, Child, Youth and Family, Child and Family Health Nursing, South Western Sydney Local Health District, Sydney, Australia; 18 Centre for Disability Research and Policy and Brain and Mind Centre, University of Sydney, Sydney, Australia; 19 The Children’s Hospital at Westmead, University of Sydney, Sydney, Australia; 20 Liverpool Hospital, South Western Sydney LHD, University of Sydney, Sydney, Australia; 21 UNSW, South Western Sydney LHD, Ingham Institute of Applied Medicine, Liverpool, Australia; 22 Infant Child and Adolescent Psychiatry, Academic Unit of Child Psychiatry, South West Sydney LHD, ICAMHS, Mental Health Centre, University of New South Wales, Liverpool Hospital, L1, Elizabeth Street, Liverpool, NSW 2170 Sydney, Australia

**Keywords:** Child development, Ecological framework, Culturally and linguistically diverse, Access, Health services

## Abstract

**Background:**

Regular health visits for parents with young children provide an opportunity for developmental surveillance and anticipatory guidance regarding common childhood problems and help to achieve optimal developmental progress prior to school entry. However, there are few published reports from Australian culturally and linguistically diverse (CALD) communities exploring parents’ experiences for accessing child health surveillance programs. This paper aims to describe and explain parental experiences for accessing developmental surveillance and anticipatory guidance for children.

**Methods:**

Qualitative data was obtained from 6 focus groups (33 parents) and seven in-depth interviews of CALD parents recruited from an area of relative disadvantage in Sydney. Thematic analysis of data was conducted using an ecological framework.

**Results:**

An overarching theme of “awareness-beliefs-choices” was found to explain parents’ experiences of accessing primary health care services for children. “*Awareness*” situated within the meso-and macro-systems explained parents knowledge of where and what primary health services were available to access for their children. Opportunities for families to obtain this information existed at the time of birth in Australian hospitals, but for newly arrived immigrants with young children, community linkages with family and friends, and general practitioner (GPs) were most important. “*Beliefs*” situated within the microsystems included parents’ understanding of their children’s development, in particular what they considered to be “normal” or “abnormal”. Parental “*choices*”, situated within meso-systems and chronosystems, related to their choices of service providers, which were based on the proximity, continuity, purpose of visit, language spoken by the provider and past experience of a service.

**Conclusions:**

CALD parents have diverse experiences with primary health care providers which are influenced by their awareness of available services in the context of their duration of stay in Australia. The role of the general practitioner, with language concordance, suggests the importance of diversity within the primary care health workforce in this region. There is a need for ongoing cultural competence training of health professionals and provisions need to be made to support frequent use of interpreters at general practices in Australia.

## Background

Early intervention for child developmental disorders has been a key health policy initiative of governments in Australia and internationally [1, 2], especially following research on early brain development [3]. It is known that optimal development in early childhood improves long-term physical, mental and emotional health. A preventative approach through anticipatory guidance is commonly incorporated into child health checks, which refer to informing parents of what to expect at the next stage of their child’s development and providing them information on issues such as nutrition, sleep and settling, behaviour management and injury prevention. This information is often provided in conjunction with regular health checks, developmental surveillance and child and family psychosocial assessment [4, 5]. There is a wealth of literature highlighting the effectiveness of these activities on improving parent-child interactions [6, 7]. However, there is limited uptake of developmental screening and surveillance, and the uptake is reduced further where children come from a culturally and linguistically diverse (CALD) backgrounds [8].

High income countries, including Australia, are increasingly diverse communities as a result of waves of immigration over the last five decades [9, 10]. A snapshot of diversity in the 2011 Australian census reflects the changes, with 15.7% of the total population born in non-English speaking countries [11], and this is much higher in the South Western (SW) region of Sydney [12]. These immigrant families are from a wide variety of cultural backgrounds, regions and countries with a multitude of spoken languages [13].

Universal primary health services are available to all permanent residents and citizens of Australia through Medicare, a government funded health insurance program [14]. However, recently arrived parents/carers from the CALD backgrounds may have limited understanding and knowledge of the NSW health care system. In addition there is the potential for hidden health vulnerabilities resulting from a lack of, or reduced health screening and immunisation provided in their country of origin [15]. Steps are being taken to address this inequity yet data is limited about Australian CALD communities and little is known about parents’ experiences of accessing developmental surveillance and primary health services for their children. Most studies and literature documenting parents’ experiences for accessing health services come from North America, where health service access for Latino and African American children has been shown to be affected by families insurance status, enrolment in Medicaid program and socio- economic variables [16, 17].

The ‘Watch Me Grow’ (WMG) longitudinal birth cohort study was conducted in the SW Sydney region to investigate the universal developmental surveillance program with the aim to generate robust evidence to inform policy and service delivery [18]. The aim of this large study was to maximise accurate early detection of children with developmental disorders through a partnership formed between policy makers, service providers and researchers. The description of the study cohort and quantitative evidence assessing the risk factors and prevalence of parental developmental concerns for their children has been reported elsewhere [19–21].

The WMG research program utilised a mixed methods study design that included a qualitative study of parents and health service providers to investigate barriers and enablers of the universal developmental surveillance program available in NSW. One aspect of the qualitative component of the WMG study is reported here, namely understanding factors influencing CALD parents’ access of primary health care services for developmental surveillance and anticipatory guidance for their children.

## Methods

### Ethics

The WMG study had ethics approvals from the South Western Sydney Local Health District and University of New South Wales (UNSW) Human Research Ethics Committees.

### Setting

The SW Sydney region of NSW is a disadvantaged area in terms of education, employment, income and occupation, as measured using a low socioeconomic index for the area (SEIFA) [22]. The population is relatively young with about 15% of residents being children 0–8 years of age [23]. The region has a large CALD population with approximately 34% of residents born overseas (some suburban areas up to 50%), and about 35% of the population has English as a second language (up to 70% in some areas). The main spoken languages other than English include Arabic, Chinese, Vietnamese, Khmer, Korean, Greek, Spanish, Italian and Serbian. Unemployment rates are high in the area (5.2 to 22.3% per cent as compared to state average of 4.7%), and the median individual incomes for over a third of the population are significantly less than the state average [24].

The primary health care system in the region has a range of service providers including child and family health nurses (CFHNs), general practitioners (GPs), paediatricians, pharmacies, and local government councils. The NSW Health Personal Health Record (PHR), known as the ‘Blue Book’ [25], is provided to parents at the time of their child’s birth, and is also available for children born interstate or overseas. The PHR includes details of the recommended developmental surveillance schedule for monitoring children’s physical and emotional development. Information and links to a range of resources and parenting support services are also found in the PHR, including access to translated versions for non-English speaking families.

### Theoretical models

Theoretical models can help us to understand how different factors interact on the path to behaviours and health outcomes [26]. An ecological model suggested by Bronfenbrenner, where the ecological environment is conceptualised as a set of nested structures, provided a framework for the investigation of parental experiences and factors that may impact their decision making in relation accessing health services for developmental surveillance and anticipatory guidance [27, 28]. The layers of structures in this framework include Micro-, Meso-, Exo-, Macro- and Chrono systems and their definitions are elaborated in Table 1. A critical element of the ecological model is *experience,* which incorporates both the objective properties of the human environment and subjective properties in the form of experience of the person living in that environment [29].

**Table 1:** Themes on parent’s experiences for accessing primary health care within the ecological framework

Ecological framework	Themes
**Microsystem:** The microsystem is a “*pattern of activities, roles, and interpersonal relations experienced by the developing person with particular physical, social, and symbolic features that invite, permit, or inhibit, engagement in sustained, progressively more complex interaction with, and activity in, the immediate environment*” [33].	**Parental concerns about their child health and development,** *‘just to know he is on track’* (Beliefs)
	**Sources of Information and support for parents, ‘** ***If I get help it’s going to be easier for me’*** (Awareness/Choices)
**Mesosystem:** The mesosystem is comprised of the relationships that exist between two or more settings [33].	**Lack of knowledge about health system and community health services, The Blue Book,** *‘Nobody told me’* (Awareness of where to access information, uncertainty of choice)
	**Choice of provider (Choices)** ***CALD parents’ understanding of the CFHN role*** ***CALD parents’ understanding of the GP role*** ***‘The importance of language’*** ***Parents’ previous experiences with CFHNs and GPs*** (Based on different factors such as proximity, continuity of care, purpose of visit, language spoken by the provider)
**Exosystem:** The exo-system refers to settings and events that influence a child’s development indirectly. This includes contexts, factors and events that may affect parents and thus impact their children.	No new themes emerged within this context
**Macrosystems:** “*The macrosystem refers to consistencies, in the form and content of lower-order systems (micro-,meso-, and exo-) that exist, or could exist, at the level of the sub-culture or the culture as a whole, along with any belief systems or ideology underlying such consistencies*” [32].	**Comparison with other health systems** Awareness and choice
**Chronosystems:** This concept acknowledges that physical and human ecologies change over time, which includes environmental events, life transitions and socio-historical events.	**Increased awareness over time** Awareness of changes to services

### Sampling and Recruitment

CALD parents were recruited from the study region using a non-probability purposeful sampling strategy to incorporate the characteristics of the population that would best answer the research question [30]. Recruitment for the study began by identifying key informants in the authors’ departments, and included nursing managers and senior community paediatricians. Co-ordinators from a Multicultural Resource Centre were subsequently contacted and were instrumental in facilitating access to multicultural supported playgroups. Participants were also recruited from Early Childhood Health Clinics. Written informed consent, including consent for audio recordings, was obtained from all participants.

### Data collection

Demographic data was collected on a standardised form. The focus groups and in-depth interviews were conducted by PG (paediatrician) and SH (CFHN), who had experience in qualitative data collection. Healthcare interpreters were used for focus groups and individual interviews for participants with minimal English, and a semi-structured interview guide was used with broad open-ended questions (Table 2). The focus groups lasted 60–90 minutes while the individual interviews lasted 15–45 minutes.

**Table 2:** Interview Guide

QNo	Question
1	If you have questions about your child’s health/development where do you go?
2	Do you regularly go for health check for your child?
3	What is your experience with the health services for your child?
4	What could have been different/better for you to go to the services?
5	What do think about the Blue Book (Personal Health Record)?

Field notes were made by the researchers and recorded data was transcribed verbatim by professional transcription services. A sub-section of the transcribed data was verified for accuracy. NVivo qualitative software was used in organising and analysing the data [31].

### Data analysis

The first cycle of data coding used hand-written notes from “line by line coding” to record recurring words or phrases. Links between the data and domains of the ecological framework were subsequently considered through a constant comparative process of what each focus group or individual stated, using a “paragraph by paragraph” approach [32]. A number of broad themes emerged in line with an ecological framework [33].

The second cycle of coding aimed to find an explanation for the themes that emerged during the first cycle of coding. An abductive inferential process was used to determine commonalities in participant responses and to understand the meaning ascribed by the participants. This served to infer the ‘best explanation’ for core underlying issues, and helped in the generation of an overarching theme. The data was ‘sieved’ repeatedly using an iterative process with consideration of alternative explanations [34].

Data collection stopped with thematic saturation. Emphasis was given to negative cases, where participants differed from the major emerging themes. For example, some parents cited no problems in accessing services for their children as compared to the dominant theme of challenges in accessing services. Two researchers coded the data independently to provide rigor to the qualitative data analysis. The second coder had no participation in the data collection, and contributed to checking the consistency of the coding. Differences in interpretation of the data were clarified by discussion between the coders and among the team members. A kappa coefficient of 0.64 and an inter-rater agreement of 92.2% showed good agreement [35].

## Results

Data was collected from a total of 40 participants from seven focus groups, with four to five participants in each group (*n* = 33), and seven individual in-depth interviews (*n* =7). All participants were mothers except for one grandparent. The focus groups varied from a group of participants from one nationality (e.g. all Vietnamese) to groups with a range of nationalities. There was also a variation in the demographic characteristics in terms of the number of years lived in Australia, the highest level of education achieved, and the current occupation reported by mothers (see Table 3).

**Table 3:** Characteristics of the participants

No	Mean Age(SD)^a^	Interpreter	Mode of data collection	Country of Birth/Origin	No of years living in Australia (Median, IQR yrs)	Education Level, (n)
IF0057-0061 & IF0041-0045	37.3(4.8)	Y	Focus group	All participants from Vietnam	9 (5–13)	Primary school-1 Yr 12- 6 Yr 10-1 UG- 2 PG- 2
IF0062-IF0065	41.7 (6.7)	N	Focus group	English Afghani Peru Vietnam	23(11–35)	Trade apprentice -2 Yr 12-1 Primary school -1
IF0029	N/A	N	Interview	Filipino	16	UG degree
IF0030	N/A	N	Interview	Australia	24	UG degree
IF0036-0040	38.2(3.6)	Y	Focus group	Vietnam-4 Cambodia-1	20 (16–26)	UG degree- 1 Yr 12- 2 PG- 1 Yr 10
IF0047-0050	34.5 (9)	Y	Focus group	All participants from Iraq	9 (6.5–10)	PG Yr 12-2 Primary school
IF0051-0055	40(11)	N	Focus group	Indian-4 Australia-1	6(5.8–13.8)	Primary school Yr 12 Trade apprentice Yr 10
IF0056	N/A	N	Interview	Afghani	12	Yr 12
IF1001	N/A	N	Interview	Caucasian	35	PG
IF1002^b^	N/A	N	Interview	Brazil	N/A	N/A
IF1003^b^	N/A	N	Interview	Arabic	N/A	N/A
IF1004^b^	N/A	N	Interview	Caucasian	N/A	N/A
IF1012-1015^b^	N/A	N	Focus Group	Caucasian	N/A	N/A

*UG* Undergraduate; *PG* Post graduate degree

^a^SD-Standard Deviation

^b^Demographic data was not available, N/A-not available

**Fig. 1:**
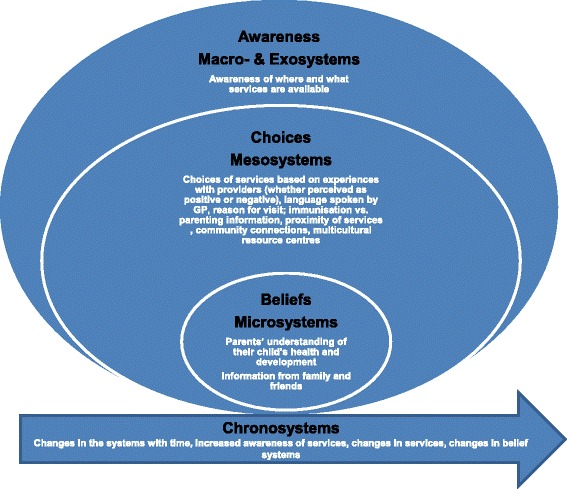
Awareness-Beliefs-Choices themes within the nested ecological framework for access to health. The figure elaborates the themes of awareness, beleifs, and choices within the Bronfennbrenner’s ecological framework of micro-, meso, macro-, exo-and chronosystems

The following section presents the overarching themes of awareness, beliefs and choices which emerged within the ecological framework, and is further highlighted in Fig. 1.

### Awareness and Beliefs

The data revealed several sub-themes in relation to CALD parents’ accessing health services for developmental surveillance and anticipatory guidance.

#### Child development: ‘Just to know he’s on track’

Firstly, the CALD parents in this study commonly mentioned some understanding about child development. As one parent said, *‘I trusted my mothers’ instinct’.* Parents and extended family members monitored if the child was achieving developmental milestones in line with their expectations. Access to services was based on their understanding of what was, and what was not, within the range of normal development. For example,

*“I’m just watching his activity at home, if it is normal. I know what’s normal, and what’s not normal…my son is 2 years old and he hasn’t started talking yet so I’m seeing a doctor. The doctor is saying that because he’s a boy his development might be slower, and it’s nothing to be worried about at this stage. I’m still worried though”.*

Secondly, parents were interested in accessing information about child development and anticipatory guidance and recognised the importance of developmental surveillance for prevention and early intervention. A Filipino mother spoke of the value of health checks incorporating developmental surveillance for her son, ‘t*hese* [health checks] *are for his future…not mine. This is for the best direction that he is going, or otherwise it’s too late to fix it’.* Another mother also indicated that it was important to know her child’s development was progressing. She was also interested in receiving information on how to promote development,

*“I know every child’s different and they need different kind of help with their development. But yeah, just to know that they’re on track, they’re going the right way. Even ideas of where to aim for next, that seems to be the biggest one—how we help him get to that place”.*

Mothers with more than one child did not feel as much need for regular health checks of their child as they considered they had an adequate understanding of child development and care. In a similar way, mothers with more ‘settled’ babies stated there was less need to access support, and those with a perceived ‘difficult infant’ sought reassurance as the primary goal of their consultation with health providers. An extra level of vulnerability was noted in first-time mothers, particularly those with limited family support, who felt challenged by their parenting responsibilities. For example, one Vietnamese mother said ‘*when I had the baby I was so scared—I didn’t know what to expect. We had all sorts of different problems like breastfeeding…so I had a very, very hard time.’* Mothers who did not have family support also seemed to have less awareness of how or where to access services; *‘because my mum was not here, so I have no family out here…it was my first pregnancy and I didn’t know where to go’.*

#### The Blue Book: *‘Nobody told me’*

A key influencing factor on parental awareness of the universal developmental surveillance program, and parenting support was the information provided to the mothers in the hospital. The mothers recalled being given the Blue Book at the birth of their baby, as per NSW health policy; however they generally did not remember time being taken to explain its use in relation to child development checks. As one mother said, *‘sometimes they forgot, I think—I stayed 2 days in hospital but couldn’t get information’* and *‘nobody told me’.* On the other hand, an Afghani mother indicated an awareness of services from information provided at the time of her baby’s birth, *‘the nurse in the hospital said if you want any help you go here, and they gave me some information.*’ This was useful to the mother when a family member indicated that there might be concerns with her child’s development; *‘I said, “No worries, we’ll go to the doctor and ask”.* An introduction to the Blue Book and its purpose by a CFHN in the community was also appreciated by a Vietnamese mother, ‘*I had my child in a different country, but when I was given the Blue Book they* [CFHN] *did explain to me that I needed to look into it….and follow up’.*

For some, the Personal Health Record was an important resource of information as well as for recording growth and development. One mother said, ‘*It’s a good thing…it shows the baby’s development—their weight, their length. They* [CFHNs and GPs] *write it down…and they had articles in there for us about what to expect from 3 to 6 months.’* However, for others it was *‘just a record for immunisation’.*

#### Other sources of information and support: *‘If I get help it’s going to be easier for me’*

CALD parents often gained information about child development from a range of sources other than health professionals. The response of one mother was indicative of many; ‘*If I find something that is slower or like not normal* [about her child’s development] *I use the internet, read books, talk to friends and get older people’s opinion’.* Mothers who had arrived in Australia more recently found it more difficult to access information about child development. For example, *‘I don’t know much about anything in this country, and it’s also hard for me to get information from the internet’.* Day Care [childcare] providers, the Multicultural Resource Centre and supported playgroups were therefore valuable resources; *‘if I can get help from any group…it is going to be easier for me’.* Parents reported seeking advice on topics such as nutrition and other anticipatory guidance, and assistance with information about services if they had concerns about their child’s development. They also indicated that support from other mothers, such as through a ‘Mothers’ Group’ was also important; ‘*…it’s a friendship because we don’t have any professionals running it… we do sort of help each other out, and it makes you feel better to know that other people are going through similar things’.*

### Choice of service provider

Parents were mostly aware there were two main providers for accessing information about their child’s development and anticipatory guidance, namely CFHNs and GPs; however they were generally unaware of the schedule for developmental surveillance. A mother from an Indian background expressed uncertainty about the most suitable health provider to access information about child development during a focus group; *‘Which one is better? We have to go to the baby clinic or GP? I don’t know’.* Some other mothers in the focus group expressed similar issues. Parents commonly made choices based on the specific purpose of their visit or the issue they wanted to discuss with the healthcare providers. Parents accessed CFHNs for information on child development, anticipatory guidance, and parenting support, while GPs were primarily utilised for immunisation and physical health concerns.

#### CALD parents’ understanding of the CFHN role

Several mothers mentioned their first contact with a CFHN was during a visit to their home through the Universal Health Home Visiting (UHHV) program following the birth of their baby. This was perceived as helpful as information was included about the developmental surveillance schedule; ‘*She* [CFHN] *gave me answers…she gave me everything. She’s explained to me, she came…like two, three times’.* Many parents recalled being asked questions about their baby’s development by a CFHN when they attended the Early Childhood Health Clinic; ‘*I found the health nurse at the clinic is more thorough in what they’re doing* [asking questions about child development and parenting support]. A Peruvian mother stated that she ‘*felt more comfortable going to a baby clinic*…*they’re dealing more with baby questions. At the GPs’ there’s a waiting room full of people…with the colds and other problems. I think the clinic is more focused on children’.* The data predominately indicated that mothers had a positive relationship with the CFHNs and valued their role. For example, *‘if I have a problem, I tell the nurse’,* and *‘…it’s important for me to talk to her* [CFHN] *and for her to embrace any concern I have…not only for that child but my other children and for myself’.*

#### CALD parents’ understanding of the GP role

Although most parents mentioned going to a GP *‘to get the immunisation’*, there were also other reasons they used the GP as the main healthcare provider for their children. Parents were confident in the knowledge of the GP; *‘we trust the GP because when they look, straight away if they observe the child has anything wrong, they mention it’* and some also chose to see a GP to maintain a stronger continuity of care;

*You couldn’t always get in to see the same nurse which is why I just started going to my GP because it was just easier. They knew what they told you last time rather than having to rely on somebody else’s notes.’*

The central role of the GP in the provision of primary health care was evident through responses such as, *‘we need a good GP who knows the family’.*

#### The importance of ‘language’

For many, a shared cultural background and language was the main factor influencing their choice of healthcare provider and particular GP; *‘sometimes you know my English is not good. And there is nowhere* [opportunity] *for discussion. If you go to the doctor, the family doctor, they all speak fluently* [the language spoken by the parents]*.* The issue of language in relation to CFHNs was not evident in the data, with limited mention of the use of healthcare interpreters that could be booked for appointments. However, some parents indicated they either preferred to communicate as best they could or with the assistance of family members who were more proficient in English.

#### Parents’ previous experiences with CFHNs and GPs

Parents’ choices were also influenced by their previous experiences of both CFHNs and GPs. Although the parents generally described positive interactions, some experiences were negative and influenced future choices. A mother with an Iraqi background felt one CFHN had an abrupt manner and the appointment ended quickly even though she ‘*still had questions to ask’*. The mother wondered if this was *‘because of my outfit or because of my scarf’*?’ The belief that the CFHN was not sensitive to her culture was the reason *‘why I don’t take her again to the same nurse’* and her choice to only go to her GP ‘*for check-ups and whatever we need to go in for’.* Time was also identified as barrier by several mothers who perceived that GPs were busy and sometimes rushed through the consultations; *‘Immunisations only—quick in, quick out…unless you ask questions* [about development] *they don’t do it’.* A Vietnamese mother made the following recommendation for both GPs and CFHNs;

*I go to the doctor, he says “Your child’s fine, they have no problem”. What I’m saying is, to the doctor and the nurse; every day is the same* [to you]*, but sometime that little bit of time and a little bit of effort, it can make a big difference to my family and myself.*

#### Other factors

In addition to issues for parents related to a limited understanding of English, other barriers were identified such as the location of their service providers and the distance they needed to travel from their homes. One mother said, ‘*The hospital gave me a paper* [information] *about a local clinic* [ECHC] *but because it’s a long drive for me, that’s why I didn’t go there.’* In addition, some parents expressed frustration with the changes in opening times and other changes for ECHCs; *‘I don’t know if they’ve closed it completely now, the baby clinic there…and then they have reduced hours so it’s hard to keep up with what days and when they were open’.*

#### Comparison with other health systems and increased awareness over time

Several parents compared health services in NSW with those in their country of origin and expressed an appreciation of the healthcare services they had encountered. A mother from Brazil said, *‘it’s so much better*…*I can’t fault anything’.* Increased awareness of services developed over time as parents became more familiar with the health system and community supports. The child of a Vietnamese parent was diagnosed with Autism Spectrum Disorder and expressed satisfaction with services as compared to her country of origin;

*When I came here, I didn’t know about any services at all, but now I’m thankful to Australia because there are a lot of services and I can access them easily. I am grateful because I compare what’s available for the children in Vietnam and children here. I have one child who’s turned three with autism....and I’m very pleased with the services.*

## Discussion

Factors influencing CALD parents’ access of primary healthcare services for developmental surveillance and anticipatory guidance emerged from the analysis of the data in this qualitative study. In relation to the ecological model, the micro-systems and meso-systems were most prominent. The CALD parents accessed information and support from extended family, and availability of services in the families’ immediate environment impacted on contact with service providers. In addition, macro-systems and chrono-systems including the impact of increasing awareness of services over time were also present. There have been three prior Australian qualitative studies exploring the issue of access of parents to health services for their children. One explored the views of parents and professionals and identified barriers to developmental surveillance; the second looked at the barriers for access for children of newly arrived refugees, while the third identified the reasons for parents accessing preventive health care for children in Melbourne [36–38]. Our study instead has focused on experiences of predominantly culturally and linguistically diverse parents, who are mainly, mixed economic and family immigrants, residing in a region of relative disadvantage in Sydney.

A key factor for CALD parents’ reduced access of primary health services was their lack of awareness of the schedule of health ‘checks’ recommended by NSW Health in the PHR (Blue Book). Overs et.al [21] found that only 46% of parents in the WMG study quantitative cohort, recalled being informed of the surveillance program, which resulted in a significant impact on the attendance and completion of the developmental surveillance tools. Missed opportunities to communicate relevant information to parents at the time of their child’s birth has been shown to widen the gaps in the continuity of care from maternity to primary health services and reduce the likelihood of participation in health visits and developmental surveillance [39, 40].

As would be expected, CALD parents were interested in knowing more about growth and development, and parenting support. Their perception of what constituted ‘normal’ child development, or the view of extended family members, informed decisions about accessing primary healthcare services in a similar way to how Canadian CALD parents of children with a disability discussed concerns with family members [41]. Although there is only limited literature examining how the parental health beliefs affect the access to primary health care for their children, a qualitative study of Vietnamese mothers in North America has shown that their traditional beliefs as such were not significant barriers for access to health services [42, 43].

Another key factor in CALD parents’ access of health visits was their choice of provider. Some parents only attended GPs for all their child health needs, whilst others used GPs for immunisation, and a CFHN for developmental surveillance, anticipatory guidance and parenting support. This is an important issue as the completion of developmental screening for infants at 6 months of age is strongly associated with attendance at CFHNs [21], whilst CALD parents in the study were less likely to access Early Childhood Health Clinics for a variety of reasons. It is known from population-based studies that mothers from non-English speaking backgrounds consult their GPs more often than CFHNs [44]. Yet the results from our study indicated that some CALD parents experienced that GPs did not ask questions about their child’s development. The difficulties faced by some parents with time constraints of their GPs is valid, as there are recent trends indicating a reduction in longer consultations provided for children at primary care practices in Australia [45]. This is particularly important as the delivery of quality primary care health service to a CALD population needs to incorporate an interpreter resulting in longer consultations [46]. Gray et al [47] has shown that an in-house interpreter was rated as most appropriate during consultations at a primary care health care centre in New Zealand when compared to the use of family members and telephone interpreters.

CALD parents preferred to visit a GP who could speak their language as this made it easier for them to discuss concerns about their child’s health. The choice of parents of a doctor from the same cultural and language background for accessing primary health services for their children has been also shown in other studies [48], as is the use of extended family members as non-professional translators [49, 50].

A positive experience of interactions with CFHNs and GPs strongly influenced the use of services, and therefore increased opportunities for developmental surveillance and anticipatory guidance. Conversely, negative experiences also impacted on parental choices. The perception of racism by an Arabic speaking mother in our study indicates a need to better understand immigrants’ experiences with day-to-day discrimination, and to consider it as a factor for healthcare service use [51, 52]. The negative experiences of some mothers may be related to issues faced by healthcare providers during service provision to CALD communities such as language barriers, inadequate assessment tools and cultural uncertainty [53]. Our study findings point to a need for healthcare providers to establish positive, non-judgmental relationships and proactively engage and sensitively communicate with CALD parents. This is likely to improve contact with CALD families [54].

CALD parents in this study indicated that community support such as through Multicultural Resource Centre programs was an important factor influencing their knowledge about child development and how to access services in the NSW Health system. The vital role of community connections by support groups and resource programs for CALD families have also been shown in other qualitative studies [41].

The overarching theme of awareness, beliefs and choices in our study finds support from the Health Beliefs Model (HBM) [55], Andersen’s healthcare utilisation model [56, 57] and principles of social psychology [58]. The HBM is primarily used for preventive health care for adults but also extends to include the health beliefs which parents have regarding their children’s health, based on their the perceived benefits and barriers to regular health checks [55]. The current study suggests that it is not only the health beliefs of the parents, but also their awareness and choices, which drive access to health visits by CALD families. These choices and beliefs of parents reflect a sense of their personal control and psychological functioning in the context of the social structures [58]. The theme of awareness, beliefs and choices further encapsulates the Andersen’s model for healthcare utilisation [56, 57, 59], by crystallising the characteristics of this model such as (a) the predisposing factors which include attitudes, values, knowledge of the health care system, social interactions and networks (awareness and beliefs), (b) the enabling factors such as the means to access services (e.g. travel times), available health facilities, and (awareness and choices) (c) the need factors, of how they view their children’s health, and what professionals have evaluated as the needs for their children (awareness-beliefs and choices).

### Implications for health services delivery

The study suggests that there is a need for improving communication about the importance of regular health checks and developmental surveillance when parents receive the PHR (Blue Book). There is a need for increasing awareness of the web based parenting and anticipatory guidance information which has been developed by the NSW Health since the conduct of the study. There is also a need for ongoing funding for migrant resource services including supported playgroups, and extend support and training of practice nurses in developmental surveillance at general practices.

There are likely gaps in the cross-cultural competence of the health care providers. There is a need to further consolidate training for professionals to promote positive interactions with CALD parents. The focus of this training should be on developing a working knowledge of the migration experience of families, acknowledging differences in child rearing and traditional cultural practices, valuing and respecting diversity, considering interpretation issues, developing a set of culture based communication skills, and developing the ability to ascertain the level of acculturation of the families [60]. The language barriers observed by the families mean that policies should also focus on the education and recruitment of culturally and linguistically diverse health care providers [61].

The GP led primary health care system is a contemporary health policy issue in Australia. The Australian government run Medicare program funds the primary health care services by reimbursing GPs the cost of consultations using a complex system of Medicare Benefits Schedule (MBS) item numbers, which takes into account the duration of consult and the type of the service which is delivered [62]. Currently, patients using GP services are either bulk billed (fully- reimbursed by Medicare) or privately billed where patients pay for their services at the fee set by the GPs incurring an out-of-pocket cost. The MBS schedule review needs to make considerations for use of interpreters by GPs for CALD populations.

There is also a need to revise and implement policies which encourage collaboration between the primary healthcare providers and support services for CALD parents. Future research on integrated models of care where GPs and CFHNs and other services are co-located has the potential to assist this process.

### Limitations

The sample population included several ethnic groups from the study region, though some minority groups were not represented due to the recruitment process and resource constraints. For example, parents from Polynesian and African communities were not included. Although not generalizable, the results corroborate findings from the quantitative component of the Watch Me Grow study, and may be transferable to other CALD populations. Interpreters were used for participants with limited English, and qualitative studies regarding use of interpreters during research process has shown that they often tend to use their own words which might ‘best’ convey the meaning of what was said by the participants, rather than providing an exact, unequivocal translation of a person’s dialogue [46, 63]. Families’ reasons for immigration, such as skilled economic migrant, refugee or international student status, were not specifically explored to maintain engagement with the participants by avoiding questions about sensitive issues. The study also did not specifically explore the experiences of mothers who have been acculturated or assimilated. In addition, factors within ecological models such as family decision making styles, specific cultural beliefs and taboos about child development, and how the support system of CALD parents impacts on access to healthcare services for children, were not explored in detail and should be the subject of further research. Despite these limitations, the main strength of the study is that the voice of CALD parents is heard, and facilitated by the culturally sensitive approach of the researchers in the data collection process.

## Conclusion

The results of this qualitative component of the ‘Watch Me Grow’ study adds to the body of knowledge about barriers and enablers to CALD parent access for information on child development and anticipatory guidance. The perspective of CALD parents informs healthcare providers and all other key stakeholders aiming to improve the long-term physical and emotional health of children by detecting developmental disorders or difficulties early.

Key findings include the importance of CALD parents gaining knowledge of where and when to access the recommended schedule of health checks. The ‘Blue Book’ and links to websites and translated information on child development should be actively promoted using social media approaches. First time parents and recent immigrants with limited contacts and awareness of services as well as low English proficiency were the most vulnerable groups for accessing health services for children. Service access was facilitated by positive experiences with service providers, and GPs who speak the first language of the mother.

The recommendation by a CALD parent is a reminder that each interaction is important and individual healthcare providers have the capacity to influence service access; ‘*that little bit of time and a little bit of effort, it can make a big difference to my family and myself’.*

## Abbreviations

CALD: Culturally and linguistically diverse
CFHNs: Child and family health nurses
ECHC: Early Childhood Health Centre
GPs: General practitioners
HBM: Health beliefs model
MBS: Medicare benefits schedule
NSW: New South Wales
PHR: Personal health record
SEIFA: Socio-economic for the area
SW: South Western
UNSW: University of New South Wales
WMG: The ‘Watch Me Grow’ study
